# Optimization of Bi_2_O_3_, TiO_2_, and Sb_2_O_3_ Doped ZnO-Based Low-Voltage Varistor Ceramic to Maximize Nonlinear Electrical Properties

**DOI:** 10.1155/2014/741034

**Published:** 2014-08-27

**Authors:** Masoumeh Dorraj, Azmi Zakaria, Yadollah Abdollahi, Mansor Hashim, Seyedehmaryam Moosavi

**Affiliations:** ^1^Material Synthesis and Characterization Laboratory, Institute of Advanced Technology, Universiti Putra Malaysia (UPM), 43400 Serdang, Selangor, Malaysia; ^2^Department of Physics, Faculty of Science, Universiti Putra Malaysia (UPM), 43400 Serdang, Selangor, Malaysia

## Abstract

In ZnO-based low voltage varistor, the two essential features of microstructure determining its nonlinear response are the formation Bi-enriched active grain boundaries as well as a controlled ZnO grain size by secondary spinel-type phases. Besides, the microstructure and phase composition are strongly affected by the dopant concentration during sintering process. In this study, the optimal dopant levels of Bi_2_O_3_, TiO_2_, and Sb_2_O_3_ to achieve maximized nonlinear electrical property (alpha) were quantified by the response surface methodology (RSM). RSM was also used to understand the significance and interaction of the factors affecting the response. Variables were determined as the molar ratio of Bi_2_O_3_, TiO_2_, and Sb_2_O_3_. The alpha was chosen as response in the study. The 5-level-3-factor central composite design, with 20 runs, was used to conduct the experiments by ball milling method. A quadratic model was established as a functional relationship between three independent variables and alpha. According to the results, the optimum values of Bi_2_O_3_, TiO_2_, and Sb_2_O_3_ were obtained 0.52, 0.50, and 0.30, respectively. Under optimal conditions the predicted alpha (9.47) was calculated using optimal coded values from the model and the theoretical value is in good agreement with the value (9.43) obtained by confirmation experiment.

## 1. Introduction

Multicomponent semiconducting ceramics that are based on ZnO and other cationic oxides exhibit highly nonlinear current-voltage characteristics [1]. ZnO varistor materials are mainly composed of ZnO as well as a balancing mix of other oxides (e.g., oxides from cobalt, nickel, bismuth, titanium, and antimony). These materials combined produce highly non-Ohmic properties and, therefore, are widely used as surge protecting elements in the electrical transmissions and circuits against lightning or temporary overvoltages [2]. With the popularity of miniaturization and integration of electronic devices, low-voltage varistors are in rapidly growing need [3]. The symmetric nonlinear current-voltage (*I-V*) response of varistor ceramics is closely related to thin insulating layers around the successive ZnO grains [4]. Microstructural studies show that the thin insulating layers around the successive ZnO grains consist of bismuth-rich second phases which promote the formation of potential barriers to electrical conduction at the ZnO homojunctions [5, 6]. Besides, secondary spinel-type phases are also located at these layers which can control the microstructure development during sintering process [7]. TiO_2_ is a spinel-forming dopant which is commonly used as a grain growth enhancing additive in the production of low-voltage ZnO-based varistor ceramics. ZnO grains' growth in the presence of TiO_2_ was explained by reduction insulating layer mobility due to the presence of spinel particles. As another spinel-forming dopant, Sb_2_O_3_ is typically added to produce fine-grained high-voltage varistor by the presence of a spinel phase at the insulating layer [8]. Each of the dopants plays a distinctive role in the subtle tuning of the final nonlinear characteristics of the varistor ceramics and cannot be omitted. Also, the proper ratios among the dopants have to be set in order to obtain the required electrical performance of the varistor ceramics through the process of microstructure development [9]. As a multivariate case, a pervious study has demonstrated that the phase equilibrium formation and as a consequence the microstructure development are strongly influenced by the TiO_2_/Bi_2_O_3_ in low-voltage varistor ceramics and the Sb_2_O_3_/Bi_2_O_3_ ratio in high-voltage varistor ceramics. This report reveals the synergistic interaction of dopant in order to achieve a desired microstructure with specific electrical properties [10]. The traditional one-factor-at-a-time approach to optimization is time-consuming and incapable of reaching a true optimum because of taking no account of comprehensive effect of factors [11]. On the contrary, the statistical experimental design allows simultaneous investigation of the effects of several process variables, as well as their actual significance on the considered response and possible interrelationship among them, giving maximum information with the fewest number of trial experiments [12–14]. For these purposes, optimization by RSM utilizing polynomial equations has been widely used [15]. This methodology requires minimum experimentation and time, thus providing to be far more effective and also cost-effective than the traditional methods of optimization [16]. In this study, RSM was used for modeling and optimizing of molar ratio of Bi_2_O_3_, TiO_2_, and Sb_2_O_3_ as additives to achieve the maximum value of the alpha for low-voltage varistor.

## 2. Experimental Procedures

### 2.1. Sample Preparation

The samples were fabricated using a conventional ceramic process with nominal composition of ZnO (99.99%), Bi_2_O_3_ (99.975%), Sb_2_O_3_ (99.6%), and TiO_2_ (99.9%) as raw powders. The raw chemicals of analytical grade produced by Alfa Aesar were weighed according to the experimental designs (Table 2) based on molar ratio [mol%]. The molar ratio was converted into weight, and electronic balance was used for precise measurement up to 10^−4 ^g. The weighed powders were mixed and milled with high-resistance zirconium balls and acetone in a polypropylene jar for 24 h. The resultant slurries were dried in oven at 100°C for 8 h. The dried pastes were crushed into powders, sieved, and pressed into pellet forms of 5.0 mm radius and 0.7 thickness at 20 MPa by Specac Hydraulic Presser machine. The green compacts were sintered at 1260°C for 2 h, with heating and cooling rates of 5°C min^−1^ [17, 18] by a box furnace (CMTS Model HTS 1400) to prepare the ceramic. To determine the electrical properties, both faces of the ceramic pellets were painted by conductive silver electrode which is called varistor.

### 2.2. Materials Characterization

The electric field-current density (*E-J*) characteristics of the varistors were recorded with Keithley 236 source meter at room temperature. The varistors were scanned by DC voltage from 0 to 100 V in step size of 2.5 V. The* I-V* data was used to calculate the alpha of the varistor by

(1) $$\begin{array}{c} \alpha =\frac{\log J_{2}-\log J_{1}}{\log E_{2}-\log E_{1}}, \end{array}$$

where *E*_1_ and *E*_2_ are the electrical fields corresponding to the current densities *J*_1_ and *J*_2_ at 0.1 and 1 mA/cm^2^, respectively. The breakdown field (*E*_*B*_) was determined at 1 mA/cm^2^ while the leakage current density (*J*_*L*_) was determined at 80% of breakdown field.

The crystalline phase compositions of the sintered sample were identified by X-ray diffractometer (XRD, PANalytical (Philips) X'Pert Pro PW3040/60, CuKα, and *λ* = 1.5418 Å) and the data were analyzed by using X'Pert High Score software. In order to make microstructural investigations, one of the surfaces of samples was ground with silicon carbide paper and polished with 1 *μ*m diamond suspension, which led to a mirror-like surface. Then, they were thermally etched at 1100°C for 10 min to reveal the microstructural details. To reduce charging effects and to improve the resolution of the image, the etched samples were metalized with a thin coating of gold mounted on Al stub. The surface microstructure and compositional analyses of the sintered samples were examined by VPSEM (LEO 1455) which is attached to EDX.

### 2.3. Experimental Design

The most popular RSM design is the central composite design (CCD) [19, 20]. A CCD has three groups of design points: (a) two-level factorial or fractional factorial design points, (b) axial points (sometimes called “star” points), and (c) center points. CCDs are designed to estimate the coefficients of a quadratic model. The factorial points, which are one unit distance away from the center of the design space, are used to fit the linear and interaction terms. The star points can provide additional levels of the factor for prediction of the quadratic terms, and their distances from the center are * α*  unit [19]. The * α*  value is equal to (2^*n*^)^1/4^, where* n* is the number of factors. In this study, *n* is equal to three effective variables, namely, the molar ratio of Bi_2_O_3_ (*A*), TiO_2_ (*B*), and Sb_2_O_3_ (*C*), so that the * α* value is 1.682.  Table 1 summarizes the ranges and levels of the effective variables in five different levels (−1.682, −1, 0, +1, and +1.682) involved in the design strategy.

Accordingly, 20 experiments which determined by consisting 8(2^*n*^) full factorial points, 6 (2n) axial points and 6 center points designed as replications to get a good estimate of experimental error (pure error). Then the design (Table 2) was performed according to the procedure in Section 2.1 and the calculated alpha (Section 2.2) is presented in column of actual value (Table 2). The completed design matrix was used for regression process by Design Expert software version 8.0.7.1 (Stat-Ease Inc., USA).

### 2.4. Statistical Analysis

The multiple regression equation was used to fit the second-order polynomial equation based on the experimental data (Table 2) as follows:

(2) Y=β0+βAXA+βBXB+βcXc+βAAXA2+βBBXB2+βCCXC2+βABXAXB+βACXAXC+βBCXBXC,

where *Y* represents the predicted response, *β*_0_ is the model intercept, *β*_*A*_, *β*_*B*_, and *β*_*C*_ are linear coefficients, *β*_*AA*_, *β*_*BB*_, and *β*_*CC*_ are quadratic coefficients, *β*_*AB*_, *β*_*AC*_, and *β*_*BC*_ are cross product coefficients, and *X*_*A*_, *X*_*B*_, and *X*_*C*_ are the independent variables influencing the response. Fitting (2) to the experimental data by the method of least squares (MLS) allowed estimation of all the coefficients [21]. The MLS is a multiple regression technique used to enquire the relationship between the independent and dependent variables and the estimator can be written as follows [22, 23]:

(3) $$\begin{array}{c} \beta ={\left(X^{\text{T}}X\right)}^{-1}X^{\text{T}}Y, \end{array}$$

where *β* is a vector of regression coefficients; *X* is an extended designed matrix of the coded levels of the input variables; and *Y* is a column vector of response determined according to the arrangements points into the experimental design.

Once *β* has been determined, it is then possible to predict *Y* and so calculate a few numbers of statistical lines of evidence whose results appear in analysis of variance (ANOVA): *F*-value, probability value (*P* value), lack of fit, coefficient of determination *R*-squared (*R*_*d*_), adjusted *R*-squared (*R*_adj_), and predicted *R*-squared (*R*_pred_). These lines of evidence confirm the quality of the fitted model by conducting Fisher's *F*-test [24, 25].

## 3. Results and Discussion

### 3.1. Model Fitting and Statistical Analysis

In the regression process, the software fitted the actual values with the polynomial equation (2) to obtain the predicted values by using RSM (Table 2). The residuals of the actual and predicted values were based on statistical analysis and model suggestion. The suggested model for the initial additives is a function of the molar ratio of Bi_2_O_3_ (*A*), TiO_2_ (*B*), and Sb_2_O_3_ (*C*) (4). Consider

(4) Y=−15.56+16.25A+25.25B+111.24C+1.18AB+6.64AC+0.4BC−21.13A2−32.06B2−194.61C2.

The results of ANOVA were used to evaluate the statistical significance of the quadratic model in general and its terms in detail (Table 3) [26] were listed in Table 3. According to the results, the model *F*-value for the model was 28.79, implying the significance of the suggested model. The regression model can explain most of the variation in the response with the large value of *F*. In addition, the associated *P* value was less than 0.05, which confirmed that this model was very significant. On the base of the results of analysis of error, the lack of fit can be achieved simply by differences between residual errors and replicated error which can provide an estimate of the pure error at the center point. A *P* value (0.1846) greater than 0.05 implies that the lack of fit is much less than the pure error which is not significant. Nonsignificant lack of fit is good and displays that the model is suitable to accurately predict the response (alpha).

**Figure 1:**
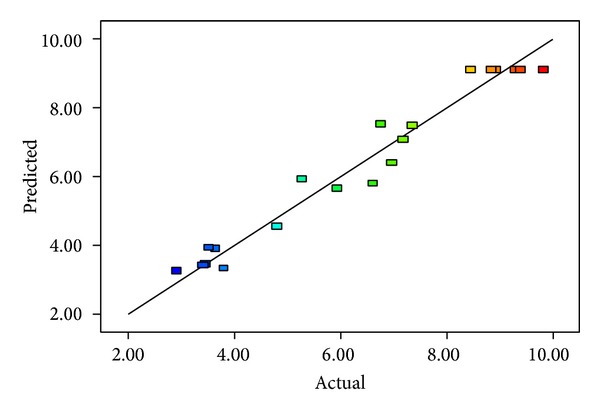
Predicted nonlinearity versus actual nonlinearity.

The *R*_*d*_ is calculated on the basis of the change in the response relative to the total variation of the response over the range of the independent factors. As shown in Figure 1, the good correlation between the observed and the predicted values (*R*_*d*_ = 0.9628) for this model indicated that this model could well explain the 96.28% of the variability in the responses. The *R*_pred_ and *R*_adj_ values should be within 0.2 of each other. The *R*_pred_ of 0.779 was in good agreement with the *R*_adj_ of 0.929 for the model. Adequate precision is a signal-to-noise ratio. Ratios greater than 4 are desirable. In the present study, a ratio of 13.279 shows that the studied model is an efficient application in the design space according to standard error.

**Figure 2:**
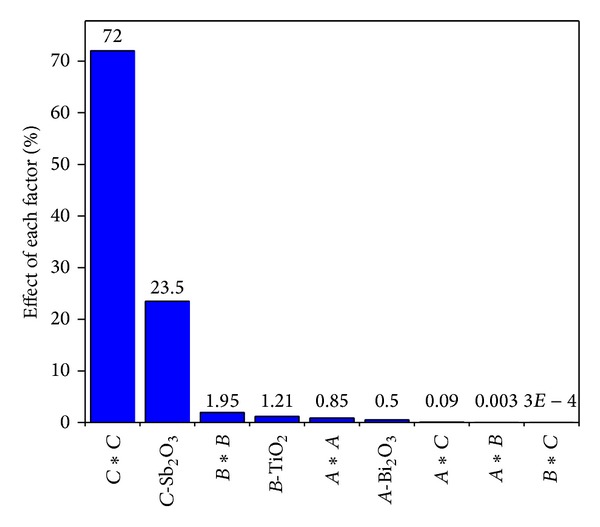
Pareto chart showing the importance of the additives and their interactions on alpha.

According to the terms of statistical analysis, Table 3 also shows the significance effect of each term in the model equation with respective *P* values. A significant *P* value (<0.05) for each term indicates an active term and a reasonable estimate of its effects. In this case, the independent variables, including *A*, *B*, *A* ^2^, *B* ^2^, and *C* ^2^, are significant model terms. In particular, the linear effect of *B*-TiO_2_ and the quadric effect of *B*-TiO_2_ and *C*-Sb_2_O_3_ are highly significant terms with *P* values <0.0001. The linear and quadric effects of *A*-Bi_2_O_3_ are significant with *P* < 0.05. In addition, none of the interaction terms had a significant effect on the response (*Y*) (*P* > 0.05). In order to determine the influence rank of each term in the model, Pareto analysis was used in the form of Pareto chart according the following equation [27]:

(5) $$\begin{array}{c} p_{i}=\left(\frac{\beta_{i}^{2}}{\sum \beta_{i}^{2}}\right)\times 100\;\left(i\neq 0\right), \end{array}$$

where *p*_*i*_ represents the percentage effect of each factor and *β*_*i*_ represents the coefficient of each term in the polynomial model. As illustrated in Figure 2, the most statistically significant variable in the model was quadratic effect of *C*-Sb_2_O_3_ (72%), followed by linear effect of *C*-Sb_2_O_3_ (23.5%). However, the antagonistic effect and synergistic effect of each variable on the response (alpha) can be determined by the negative and positive signs of regression coefficients in the model equation.

### 3.2. RSM Analysis

**Figure 3:**
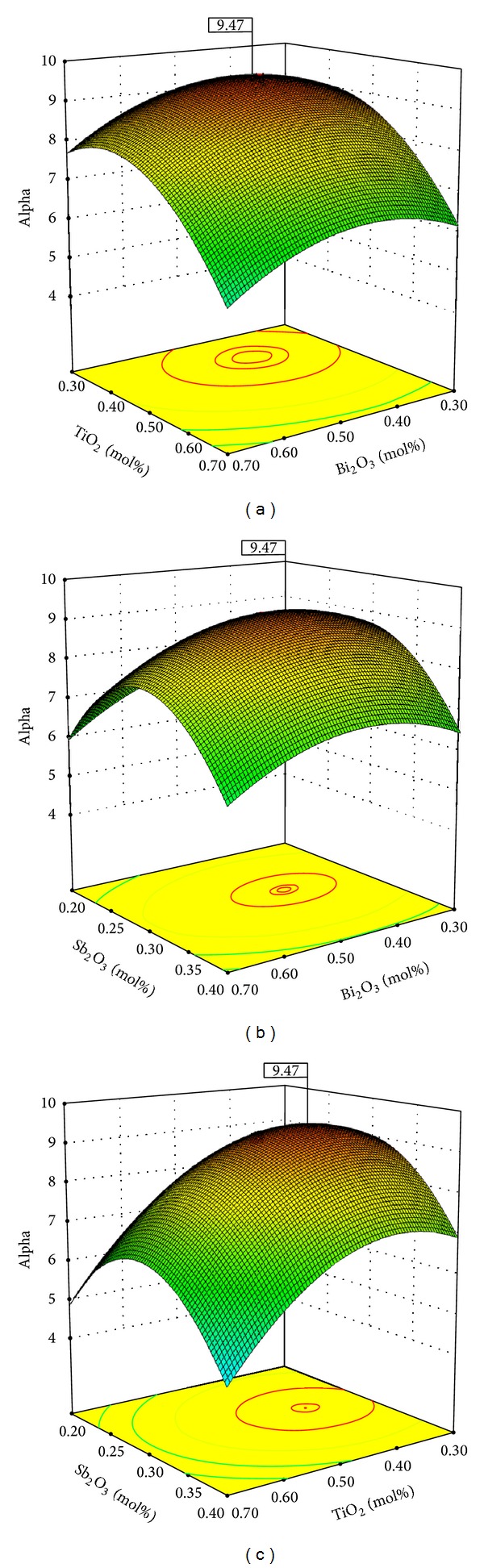
Three-dimensional surfaces plot: (a) effects of Bi_2_O_3_ and TiO_2_ on the alpha (Sb_2_O_3_ = 0.29 mol%); (b) effects of Bi_2_O_3_ and Sb_2_O_3_ on the alpha (TiO_2_ = 0.4 mol%); (c) effects of TiO_2_ and Sb_2_O_3_ on the alpha (Bi_2_O_3_ = 0.44 mol%).

In order to better understand the relationship between the response (alpha) and the independent variables *A*, *B*, and *C*, three-dimensional surfaces plots were formed based on the model polynomial function (Figure 3). Meanwhile, the maximum achievable alpha point was determined by the partial derivative of model (4) with respect to its variables *A*, *B*, and *C* as presented in following equations:

(6) $$\begin{array}{c} {\left[\frac{\partial Y}{\partial A}\right]}_{BC}=0,\\ {\left[\frac{\partial Y}{\partial B}\right]}_{AC}=0,\\ {\left[\frac{\partial Y}{\partial C}\right]}_{AB}=0. \end{array}$$

By solving the system of (6), the maximum achievable alpha point was found to be 9.47. The corresponding parameters that yielded this maximum value are *A*-Bi_2_O_3_ (0.44 mol%), *B*-TiO_2_ (0.4 mol%), and *C*-Sb_2_O_3_ (0.29 mol%).

Figure 3(a) shows the combined effect of variables *A* and *B* on alpha at constant molar ratio of Sb_2_O_3_ (0.29). As shown, the alpha increased with increasing molar ratio of Bi_2_O_3_ between 0.3 and 0.44 and TiO_2_ between 0.3 and 0.4; however, with further increase more than the optimum (Bi_2_O_3_: 0.44 mol% and TiO_2_: 0.4 mol%), the alpha decreased.

Figure 3(b) signifies the impact of changing amount of Bi_2_O_3_ and Sb_2_O_3_ on the alpha while the amount of TiO_2_ was fixed at 0.4. As shown in the figure, it is clear that when the molar ratio of Bi_2_O_3_ and Sb_2_O_3_ increases, the alpha increases firstly and then decreases after the maximum point (9.47).

The combined effect of the amount of TiO_2_ and Sb_2_O_3_ has been presented in Figure 3(c) while the amount of Bi_2_O_3_ was kept constant at 0.44 mol%. The results show that the maximum alpha (9.47) was recorded at 0.4 and 0.29 mol% of TiO_2_ and Sb_2_O_3_, respectively.

In all plots, addition of Bi_2_O_3_ up to the optimum points increases the non-linear coefficient (alpha) of the varistor ceramics. This shows that the bismuth content increases in the ZnO samples, someway advocating the cationic interdiffusion and the mass transport processes throughout the sintering process. It is well identified that the majority of bismuth-doped ZnO varistors have been made ready through liquid phase sintering process. On the other hand, the growth of the Bi_2_O_3_ content leads to a growth of the potential barrier height, causing an increase in the value of alpha [28]. However, further increase beyond optimum points might cause the homogeneous segregation of the additives, causing a decline in the level of alpha [29].

As can be seen in all 3D plots with increasing amount of TiO_2_ up the optimum points, the nonlinear exponents have been enhanced. According to a previous report, TiO_2_ increases reactivity of the Bi_2_O_3_-rich liquid phase with the solid ZnO throughout the sintering process which avoids Bi_2_O_3_ vaporization, probably leading to increase in the nonlinear coefficient [18]. However, with more TiO_2_ doped (more than optimum points), the dopants will worsen the nonlinear electrical properties of the samples. This might be linked to the sudden reduction of the quantity of Bi_2_O_3_ in the varistor ceramics owing to the reaction between Bi_2_O_3_ and TiO_2_ into secondary phases, consuming out the varistor-forming oxide. It was stated that, throughout sintering, Bi_2_O_3_ provides for the development of insulating boundary layers that regulate the varistors operation [30].

As shown in all plots, the alpha increased by increasing the amount of Sb_2_O_3_ below the optimum points. The increasing of the amount of antimony oxide favors the densification of the ceramic matrix during the sintering process of ZnO-based varistors. Moreover, the higher concentration of antimony also shows a significant role in increasing the grain boundary resistivity, as it heightens the probability of segregation forming effective potential barriers and consequently increasing the level of alpha [31]. However, with more increasing beyond optimum levels the alpha values decreased. This reduction may be attributed to the decrease in the amount of Bi_2_O_3_ since Bi is incorporated into spinel particles till Bi_2_O_3_ ultimately fades as the amount of Sb_2_O_3_ enhanced [32].

### 3.3. Confirmation Experiment

On the basis of RSM, the optimum values of the test variables were Bi_2_O_3_, 0.44 mol%; TiO_2_, 0.4 mol%; and Sb_2_O_3_, 0.29 mol%. Under these conditions, the maximum predicted alpha was 9.47. The verification experiment was carried out by studying under optimal conditions. An actual value of 9.43 ± 0.42 was observed from real experiments, which are in close agreement with the model predicted values (9.47). The results (Table 4) indicated that verification study confirmed the predictivity of the model.

### 3.4. Structural and Morphological Properties of Validated Varistor

**Figure 4:**
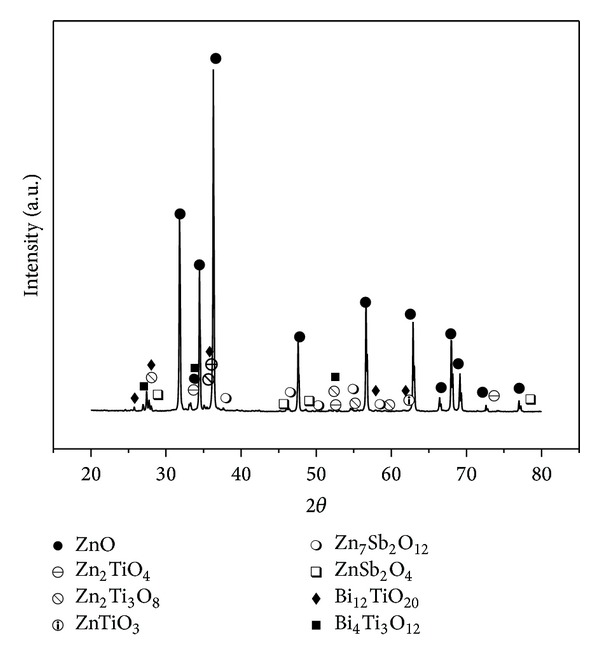
XRD patterns of the ceramic core of ZnO validated varistor made at the predicted conditions.

Figure 4 represents the XRD spectrum of the validated polycrystalline ceramic used as core in ZnO-based low-voltage varistor. The patterns confirmed the presence of dominant ZnO phase (ICSD code: 00-005-0664) with hexagonal wurtzite structure and secondary phases. Many secondary phases with small peaks were detected in the ceramics at all sintering temperatures, namely, Bi_4_Ti_3_O_12_ (ICSD code: 00-008-0258), Bi_12_TiO_20_ (ICSD code: 00-034-0097), Zn_2_TiO_4_ (ICSD code: 00-025-1164), Zn_2_Ti_3_O_8_ (ICSD code: 00-013-0471), ZnTiO_3_ (ICSD code: 00-026-1500)), Zn_7_Sb_2_O_12_ (ICSD code: 00-036-1445), and ZnSb_2_O_4_ (ICSD code: 00-004-0563).

**Figure 5:**
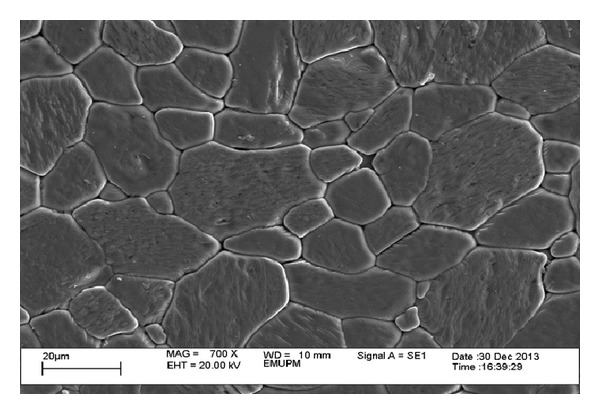
The microstructure of the validated varistor morphology.

**Figure 6:**
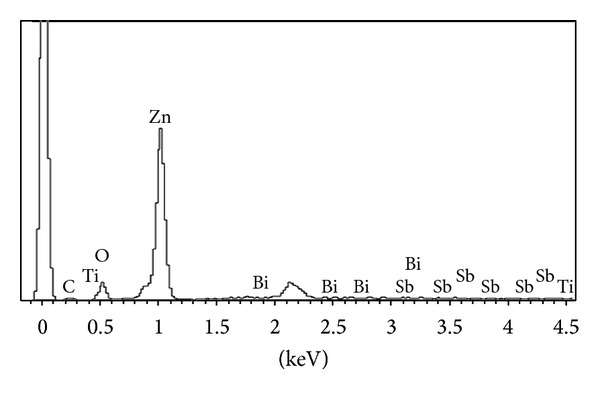
The EDX of etched validated varistor surface.

The morphology and microstructure of the sample fabricated under the optimum conditions were studied by VPSEM (Figure 5). As shown, the grains were uniformly distributed throughout the microstructure of sample with nearly no pore which resulted the high relative density and consequently the reliable non-linear electrical property. From EDAX analysis, the Bi, Ti, and Sb were found at the grains boundaries (Figure 6).

The electrical properties of the varistor were basis of* I-V* characteristic measurement that shows breakdown voltage was 120 V/mm with alpha 9.43. The leakage current was 0.013 mA/cm^2^.

## 4. Conclusion

In this study, low-voltage Bi_2_O_3_-TiO_2_-Sb_2_O_3_ doped ZnO varistor ceramics have been successfully prepared by conventional fabrication technique. The RSM was used to optimize the molar ratio of Bi_2_O_3_, TiO_2_, and Sb_2_O_3_ as additives in starting powder and a second-order polynomial equation was developed for describing the influence of key variables on response (alpha). The results of model fitting and statistical analysis demonstrated that variables *A*, *B*, *A* ^2^, *B* ^2^, and *C* ^2^ played a key role in the response, while the linear effect of *C* and interactions of *AB*, *AC*, and *BC* demonstrated a negligible effect on the response. The optimal conditions for the response (alpha) were found as Bi_2_O_3_ of 0.44 mol%, TiO_2_ of 0.40 mol%, and Sb_2_O_3_ of 0.29 mol%. The predicted alpha value determined as 9.47 under the optimal conditions and the verification study (9.43) confirmed the suitability of the predicted model. As a conclusion, RSM through the CCD model is appropriate for determining the optimal conditions for alpha, understanding the relationships among the independent and response variables, and maximizing the alpha.

## Figures and Tables

**Table 1:** Experimental ranges and levels of the effective variables in initial ceramic powders.

Symbol	Variable	Unit (%)	Level of variables				
			−1.682	−1	0	1	+1.682
*A*	Bi_2_O_3_	mol	0.16	0.3	0.5	0.7	0.84
*B*	TiO_2_	mol	0.16	0.3	0.5	0.7	0.84
*C*	Sb_2_O_3_	mol	0.13	0.2	0.3	0.4	0.47

**Table 2:** The five-level experimental design of the three additives, Bi_2_O_3_, TiO_2_, and Sb_2_O_3_, as ceramic initial powders used in ZnO-based low-voltage varistor. The data was presented by coded and actual values.

Run	Type	Coded variable			Actual variable			*Y* (alpha)	
		*A*	*B*	*C*	Bi_2_O_3_	TiO_2_	Sb_2_O_3_	Actual value	Predicted value
1	Factorial	−1	−1	−1	0.3	0.3	0.2	7.19	7.10
2	Factorial	+1	−1	−1	0.7	0.3	0.2	6.62	5.82
3	Factorial	−1	+1	−1	0.3	0.7	0.2	4.82	4.55
4	Factorial	+1	+1	−1	0.7	0.7	0.2	3.45	3.46
5	Factorial	−1	−1	+1	0.3	0.3	0.4	6.97	6.42
6	Factorial	+1	−1	+1	0.7	0.3	0.4	5.95	5.67
7	Factorial	−1	+1	+1	0.3	0.7	0.4	3.65	3.90
8	Factorial	+1	+1	+1	0.7	0.7	0.4	3.80	3.34
9	Axial	−1.682	0	0	0.2	0.5	0.3	7.36	7.49
10	Axial	+1.682	0	0	0.9	0.5	0.3	5.29	5.94
11	Axial	0	−1.682	0	0.5	0.2	0.3	6.77	7.53
12	Axial	0	+1.682	0	0.5	0.9	0.3	3.41	3.43
13	Axial	0	0	−1.682	0.5	0.5	0.1	3.51	3.94
14	Axial	0	0	+1.682	0.5	0.5	0.5	2.91	3.26
15	Central	0	0	0	0.5	0.5	0.3	8.93	9.10
16	Central	0	0	0	0.5	0.5	0.3	9.30	9.10
17	Central	0	0	0	0.5	0.5	0.3	9.39	9.10
18	Central	0	0	0	0.5	0.5	0.3	9.83	9.10
19	Central	0	0	0	0.5	0.5	0.3	8.85	9.10
20	Central	0	0	0	0.5	0.5	0.3	8.46	9.10

**Table 3:** ANOVA of quadratic model.

Source	Sum of squares	Degree of freedom	Mean square	*F*-value	*P* value	Suggestion
Model	100.24	9	11.14	28.79	<0.0001	Significant
*A*	2.89	1	2.89	7.47	0.0211	—
*B*	20.30	1	20.30	52.46	<0.0001	Significant
*C*	0.55	1	0.55	1.41	0.2623	—
*AB*	0.018	1	0.018	0.046	0.8346	—
*AC*	0.14	1	0.14	0.37	0.5590	—
*BC*	5.2*E* − 004	1	5.2*E* − 004	1.3*E* − 003	0.9715	—
*A* ^2^	10.30	1	10.30	26.62	0.0004	Significant
*B* ^2^	23.70	1	23.70	61.24	<0.0001	Significant
*C* ^2^	54.58	1	54.58	141.06	<0.0001	Significant
Residual	3.87	10	0.39			
Lack of fit	2.72	5	0.54	2.35	0.1846	Not significant
Pure error	1.15	5	0.23			
Corrected total	**104.11**	**19**				

*R*-squared		0.9628	Standard deviation		0.62	
Adjusted *R* ^2^		0.9294	Coefficient of variation %		9.84	
Predicted *R* ^2^		0.7793	PRESS		22.98	
Adequate precision		13.279				

**Table 4:** The model experimental predicted and validated values of alpha at the prediction conditions.

	Bi_2_O_3_ (% mol)	TiO_2_ (% mol)	Sb_2_O_3_ (% mol)	Alpha
Predicted condition	0.44	0.4	0.29	9.47 (predicted)
Validated condition	0.44	0.4	0.29	9.43 ± 0.42 (actual)
